# Modeling to capture bystander-killing effect by released payload in target positive tumor cells

**DOI:** 10.1186/s12885-019-5336-7

**Published:** 2019-03-04

**Authors:** Jong Hyuk Byun, Il Hyo Jung

**Affiliations:** Department of Mathematics, Pusan National University, Busan, 46241 South Korea

**Keywords:** Bystander-killing effect in ADCs, Antibody-drug conjugates (ADCs), Dose-response curve, Age-structure model, Tumor growth inhibition (TGI) model

## Abstract

**Background:**

Antibody-drug conjugates (ADCs) are intended to bind to specific positive target antigens and eradicate only tumor cells from an intracellular released payload through the lysosomal protease. Payloads, such as MMAE, have the capacity to kill adjacent antigen-negative (Ag–) tumor cells, which is called the bystander-killing effect, as well as directly kill antigen-positive (Ag+) tumor cells. We propose that a dose-response curve should be independently considered to account for target antigen-positive/negative tumor cells.

**Methods:**

A model was developed to account for the payload in Ag+/Ag– cells and the associated parameters were applied. A tumor growth inhibition (TGI) effect was explored based on an ordinary differential equation (ODE) after substituting the payload concentration in Ag+/Ag– cells into an Emax model, which accounts for the dose-response curve. To observe the bystander-killing effects based on the amount of Ag+/Ag– cells, the Emax model is used independently. TGI models based on ODE are unsuitable for describing the initial delay through a tumor–drug interaction. This was solved using an age-structured model based on the stochastic process.

**Results:**

*β*∈(0,1] is a fraction parameter that determines the proportion of cells that consist of Ag+/Ag– cells. The payload concentration decreases when the ratio of efflux to influx increases. The bystander-killing effect differs with varying amounts of Ag+ cells. The larger β is, the less bystander-killing effect. The decrease of the bystander-killing effect becomes stronger as Ag+ cells become larger than the Ag– cells. Overall, the ratio of efflux to influx, the amount of released payload, and the proportion of Ag+ cells determine the efficacy of the ADC. The tumor inhibition delay through a payload-tumor interaction, which goes through several stages, may be solved using an age-structured model.

**Conclusions:**

The bystander-killing effect, one of the most important topics of ADCs, has been explored in several studies without the use of modeling. We propose that the bystander-killing effect can be captured through a mathematical model when considering the Ag+ and Ag– cells. In addition, the TGI model based on the age-structure can capture the initial delay through a drug interaction as well as the bystander-killing effect.

**Electronic supplementary material:**

The online version of this article (10.1186/s12885-019-5336-7) contains supplementary material, which is available to authorized users.

## Background

ADCs are complex engineered compounds consisting of a monoclonal antibody (mAb), cytotoxic payload, and linker connecting the mAb and payloads [1]. In addition, mAbs are responsible for a specific target (Ag+) in the tumor cells. Payloads are potent drugs for the killing of tumor cells. Emtansine [2] and MMAE [3] are examples of substances used in the treatment of breast cancer and Hodgkin’s lymphoma or anaplastic large cell lymphoma, respectively [4]. The mechanism of ADCs [5] can be described as follows: The mAbs bind to a specific target on the surface of the tumor cells and are effectively internalized through endocytosis. Payloads are then released into the cytosol as a result of the lysosomal protease, followed by their binding to tubulin to prevent microtubule polymerization, which results in tumor death. It is known that the released MMAE in Ag+ cells enters the target negative (Ag–) cells, which do not directly interact with the ADCs, based on the membrane permeability [6]. This is called the bystander-killing effect, which induces an additional tumor reduction.

A compartment system [7] comprises of a finite number of compartments, each of which is homogeneous and well mixed, interacting through the exchange of materials. However, tumors exist in a heterogeneous manner [8] and are a mixture of various types of cells. This obscures the modeling of the interaction between the tumor cells and administered drug. To simplify such complicity, two homogenous tumor cells are considered, one of which is Ag+ cells, and the other is Ag– cells. This study proposes that the tumor growth of Ag+/Ag– cells should be treated independently. Otherwise, the modeling considering two types of cells makes no difference after a proper time scale is applied, upon which the model becomes meaningless. To support this idea, we developed a model to account for the released payload in Ag+ cells trickling into Ag– cells with suitable tumor proportions. The amount of intracellular-released payload in the tumor cells is known to determine the ADC efficacy [6]. We observed that the ADC efficacy also depends on the ratio of influx/efflux, from the intercellular to the extracellular spaces, and the amount of Ag+ cells among all of the cells. This study surveyed such reasons through a mathematical model. The other main purpose is to capture the bystander-killing effect. Considering two types of cells, the change in the total tumor sizes through the bystander-killing effect was investigated according to the ratio of Ag+ to Ag– cells. Meanwhile, because exponential/logistic tumor growth models are not applicable owing to a lack of description regarding the tumor inhibition delay by drug-tumor cell interaction, we suggest an age-structured model to elucidate the delay. Consequently, the model formulates that the amount of intracellular-released payload, the influx-to-efflux ratio, and the amount of Ag+ cells among all of the cells determine the ADC efficacy and demonstrate the bystander-killing effect.

## Methods

### Emax and TGI models

An Emax model (or function) [9] describes a dose-response curve governing the binding of a drug to a target antigen based on the law of mass action. Biologically, the response over the amount of drug dose reaches a maximum *E*_*max*_ similar to the Michaelis-Menten kinetics [10]. The Emax model *E* for a response inhibition by the applied drugs is given by

$$ E=E_{0}\left(1-\frac{E_{max}c^{\gamma}}{IC_{50}^{\gamma}+c^{\gamma}}\right), $$

where *E*_0_ is the response predicted when the released payload is zero, *E*_*max*_ is the maximum killing effect, *IC*_50_ is the concentration when the effect of the concentration reaches *E*_*max*_/2, and the hill coefficient *γ* is a cooperative or sigmoid coefficient. The TGI model is used for a tumor reduction based on the drug administration [11]. The model reads as follows:

1 $$ \frac{dT}{dt}=cEF(t)T(t)-\lambda T(t),  $$

where *F*(*t*)=1 or 1−*T*/*T*_*max*_. We additionally add a logistic growth, which will be discussed later.

### Modeling of payloads considering Ag+ and Ag– cells

**Fig. 1:**
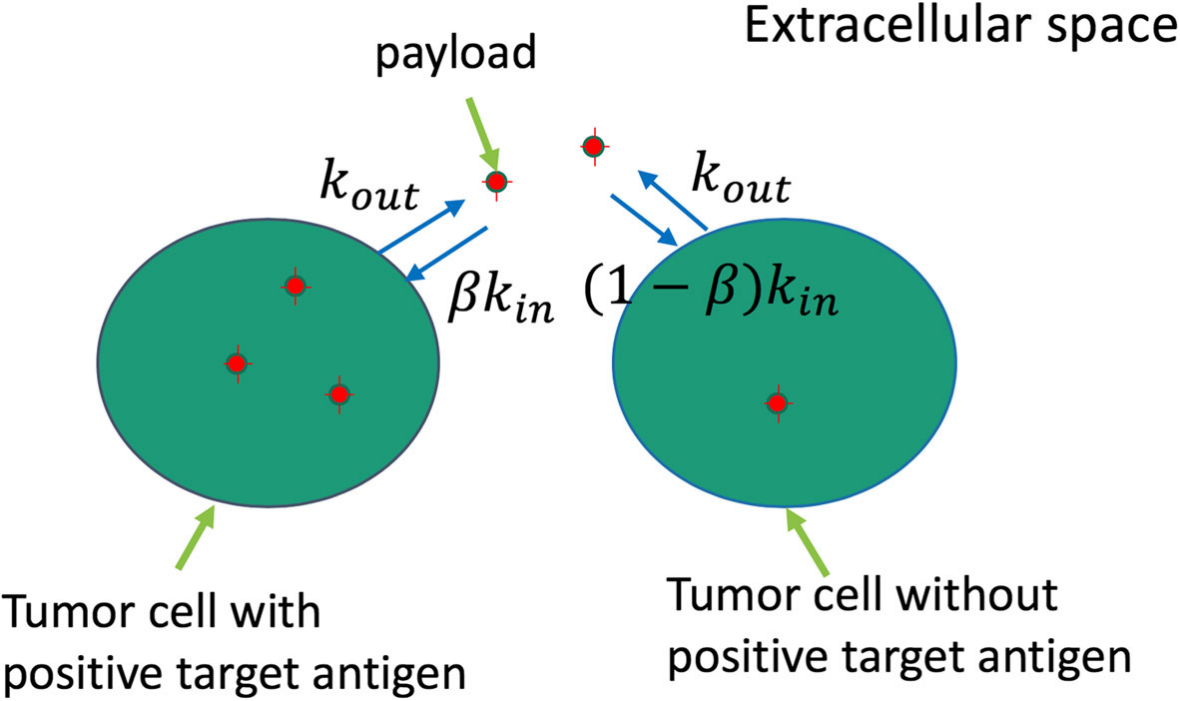
Schematic diagram. The payload in cytosol trickles out into the extracellular space and reenters into the cytosol. Some of the extracellular-released payload enters into the Ag– cells, which results in a bystander-killing effect

Tumor cells consist of target positive/negative antigens in terms of the mAbs. In such a heterogeneous manner, the change in the total tumor size over time will be investigated. That is, the payload concentration of Ag+ cells diffusing to Ag– cells is explored. The translated payload concentration may trigger an additional TGI effect in Ag– cells. We are not concerned with the mechanism, such as ADC dynamics, that represents the ADC-target interaction, because doing so makes the system more complex, and many parameters need to be estimated. A drawback here is that the ADC dynamics are not considered, nor is the direct payload diffusivity to the tumor. Our study describes the degree of bystander-killing through the model when a direct administering of drugs with bystander-killing is applied. In vitro experiments were assumed by not considering the clearance within the extracellular space. Thus, we studied the payload in intracellular diffusion into Ag– cells and how it triggers the bystander-killing effect. Because a fraction *β*∈(0,1] is considered, the proportion of Ag+ cells among all tumor cells will be determined. For example, if *β*=2/3, then the tumor cells consist of 70% Ag+ and 30% Ag– cells. Assume that the payload is only administrated once in the Ag+ cells, and the payload in Ag+ is expressed by *C*_*int,p*_. The payload concentration in the Ag– cells is given using *C*_*int,n*_, and *C*_*ext,p*_ is the payload concentration in an extracellular space. In the model, we do not regard the increasing payload concentrations, which cause ADC cleavage to occur during binding or circulation through phagocytes and cathepsin B. Therefore, we only reflect the case in which the linker is broken in the lysosome after the internalization of the ADC, and the payload concentration then increases. Considering this, the following system of ODEs can be considered.

2 $$ \begin{aligned} \frac{dC_{int,p}}{dt}=&\beta k_{in}C_{ext,p}-k_{out}C_{int,p}\\ \frac{dC_{int,n}}{dt}=&(1-\beta) k_{in}C_{ext,p}-k_{out}C_{int,n}\\ \frac{dC_{ext,p}}{dt}=&- k_{in}C_{ext,p}+k_{out}C_{int,p}+k_{out}C_{int,n}, \end{aligned}  $$

where *k*_*in*_ and *k*_*out*_ are the influx and efflux rates, respectively. A schematic diagram is shown in Fig. 1. Because the system of ODEs is linear, it can be solved explicitly.

Some parameter values are known. These parameter values are derived from mAbs, such as Herceptin, and ADCs, including brentuximab-vedotin and T-DM1, and may vary depending on the experimental environment [12–16]. Based on a particular study [16], the payload influx/efflux rate *k*_*in*_ and *k*_*out*_ were deemed to be 8.46·10^−2^ and 4.122·10^−2^ per minute, respectively. The values are at a day-scale of approximately 121.824 and 5.9357·10^4^. The ratio, *k*=*k*_*out*_/*k*_*in*_, is investigated using 1, 2, and 3 to examine the influence of the tumor reduction. In addition to applying the TGI model, *IC*_50_=300 *nM* from [12], *E*_0_=120 *nM* from [16], and the initial tumor size *T*_0_=1000 *mm*^3^ are chosen. The maximum killing rate *E*_*max*_=0.6931 is from [17]. The tumor growth rate, represented by *c*·*E*_0_, is properly determined for a comparison with the exponential tumor growth when the payload is not injected. The hill constant *γ* influences the stiffness of the TGI curve, and we assume *γ*=1 in the present study. The degradation rate *λ* is assumed to be 0.5 per day. The initial condition in (1) is considered as follows: From the initial total tumor size *T*_0_, the Ag+ cells/Ag– cells are divided by the value *β*, i.e., if *β*=1/4, then the positive target tumor cell size is 1/4·*T*_0_, and the negative target tumor cell size is 3/4·*T*_0_. Thus, the initial tumor size in the Ag+ cell is *β*·*T*_0_. The initial payload concentrations *C*_*int,p*_(0),*C*_*int,n*_(0), and *C*_*ext,p*_(0) are chosen to be 200 *nM*, 0, and 0, respectively. The gamma probability density function, which has a skewed bell-shape curve, may be used to demonstrate the payload dose heuristically, but ADC disposition in plasma, ADC-target interaction, and ADC-target complex, ADC diffusion into tumors should be added in the model for better clinical setting.

## Results

We first examine the case without a payload administration. If no payload is injected, then the tumors grow exponentially if *F*=1. From this, *c* is properly chosen to be 4.6·10^−3^ per day. Thus, the tumor growth rate is *c*·*E*_0_=0.552, which is compared to the degradation rate *λ*=0.5. Conveniently, *k* uses 2 instead of 2.0442, which is from *k*_*out*_/*k*_*in*_=(5.9357·10^4^)/121.824. Because *k*_*in*_ is too fast, it is difficult to capture the payload dynamics at the initial time, and we thus assume *k*_*in*_=1. Here, *F* is used as the logistic growth without comment. The logistic TGI model is considered along with the drug-tumor model [11] and the logistic tumor model [18]. In this case, the maximum tumor size *T*_*max*_ is assumed to be 2·10^4^ after several trials.

### Change in tumor cell growth using the total payload

The TGI model is used to investigate the delay in the tumor growth by substituting the total payload *C*_*int,p*_+*C*_*int,n*_ into *E*, in which the initial payload *C*_0_=200*nM* is used. Although the values of *β* under a fixed *k* are varied, a difference in tumor delay is not observed. This is because the total payload *C*_*int,p*_+*C*_*int,n*_ is independent on *β* owing to *d*(*C*_*int,p*_+*C*_*int,n*_)/*dt*=*k*_*in*_*C*_*ext,p*_−*k*_*out*_(*C*_*int,p*_+*C*_*int,n*_). Thus, the values of *β* become regardless of the tumor reduction. This indicates that the model is not valuable if the total concentration is substituted into *E*. In the present study, $E=E_{0}\cdot (1-E_{max}(C_{int,p}+C_{int,n})^{\gamma })/(IC_{50}^{\gamma }+(C_{int,p}+C_{int,n})^{\gamma })$ by the total payload will not be used for determining the influence of the Ag+/Ag– cells.

### Influences of *C*_0_, *k*, and *β*

**Fig. 2:**
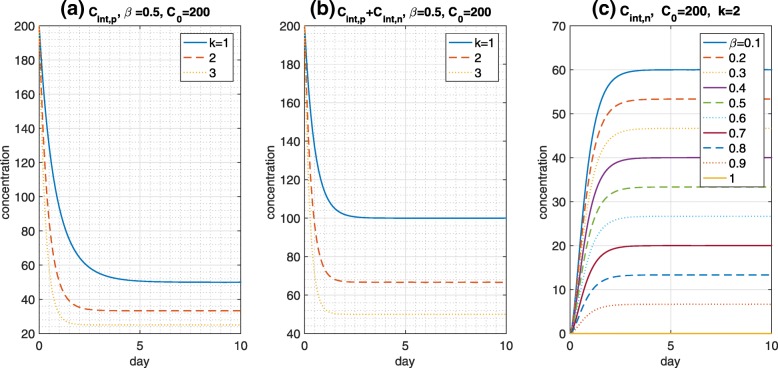
Payload dynamics. (**a**) As *k* decreases, the payload concentration in the Ag+, Ag–, and all cells increases, as shown in (**a**) and (**b**). For a fixed *k*=2, the payload concentration over time in the Ag+/Ag– cells is plotted with variations in *β* in (**c**)

Here, initial payloads *C*_0_=0, 10, 100, and 200 nM are applied to the model. The initial payload concentrations in the intracellular space are considered a case of direct injection into the tumors, rather than determining the payload concentration through the dosing process. This is clear if *C*_0_ increases, and the tumor (growth) delay is stronger. In addition, *k*=*k*_*out*_/*k*_*in*_ under a fixed *k*_*in*_=1 is investigated using values of 1, 2, and 3. If *k* is smaller, then the tumor delay is strengthened. That is, a decrease in *k* causes a stronger tumor reduction because the payload concentration is slowly released into the extracellular space by decreasing the efflux rate *k*_*out*_, which results in a higher payload concentration in the tumor cells. Thus, the tumors will be more suppressed. In addition, a small change in k may trigger both tumor (growth) suppression and tumor stimulation. That is, if we consider the exponential growth in the TGI model, from *dT*/*dt*=(*cE*−*λ*)*T*(*t*), tumors will increase if *cE*>*λ*, and decrease otherwise. An increase in payload depends on a decrease in *k*, followed by a decrease in *E*, and vice versa. We thus choose a suitable $\tilde k$ among *k*’s such that, for the given $\epsilon >0, c(\tilde E-\epsilon)<\lambda $, and $c\tilde E>\lambda $ within a particular time interval. Here, *β* determines the amount of Ag+/Ag– cells, and an increase in *β* causes an increase in the payload concentration in Ag+ cells, but not in Ag– cells. The total payload concentration, which is independent of *β*, converges to a point below the initial *C*_0_ after a certain amount of time has passed based on the extracellular payload. Meanwhile, it is unclear how each Ag+/Ag– cell is suppressed by *β*. blackIn Fig. 2a and b, the payload concentration in Ag+/Ag– cells increases as *k* decreases. In addition, the values of *β* determine the payload concentration in each cell. The payload concentration increases as *β* decreases in Ag– cells, contrary to the Ag+ cells shown in (c). For example, *β*=0.7 implies that the size of the Ag+ cells is 700 *mm*^3^, and the payload concentration converges at approximately 50 nM, at which the payload in Ag– cells is approximately 20 after 2 days. Thus, the total payload is approximately 70 *nM*, as shown in Fig. 2b and c.

### Independent Ag+/Ag– cell growth

**Fig. 3:**
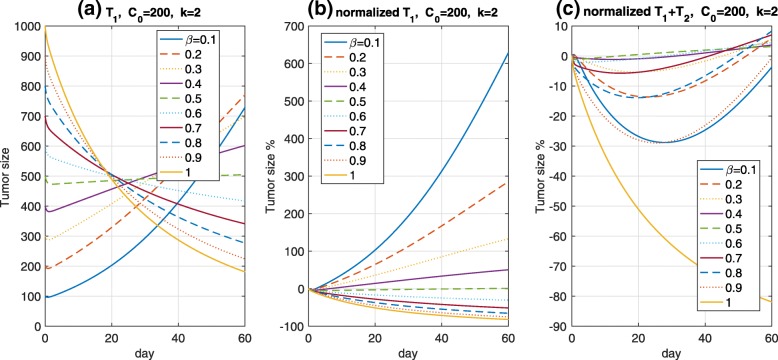
Tumor dynamics. The values of *β* were investigated to examine changes to each tumor size in (**a**). The legend shows the ratio of Ag+ to Ag– cells. Here, *T*_1_,*T*_2_, and *T*_1_+*T*_2_ represent Ag+, Ag–, and total tumor cell sizes, respectively. Each *T*_1_ and *T*_2_ appear complex owing to the different initial sizes, although a normalized form may elucidate the differences among *β*’s, as shown in (**b**). We also show the percentage of changes in the total tumor sizes in (**c**)

Tumor cells present in a heterogeneous manner, and show a mixture of several types of cells; however, we have considered two different types of cells, and the total cells are assumed to be homogeneously separated into two regions, as previously mentioned. In a simple case, we suppose that the separated cells grow independently. This motivated an investigation into the influence of the value of *β*. The TGI model is used to observe the tumor-growth delay of each cell, one of which is *T*_1_/ *T*_2_ representing Ag+/Ag– cells, respectively. The total tumor size *T*_1_+*T*_2_ is considered to account for the values of *β* and the tumor reduction. The parameter values are same as the fixed *k*=2, and TGI models, both exponential and logistic, are used; however, the results are only plotted in the logistic growth model, although both models show similar dynamical behaviors with the exception of the tumor sizes. We examined the total tumor reduction using *β*s when the tumors were suppressed independently by the payload. In addition to each tumor size based on *β*, normalization for a comparison of the Ag+/Ag– cell sizes is conducted, namely, 100·(*T*_*i*_/*T*_*i*,0_−1), where *T*_*i*_, *i*=1,2 are the Ag+ and Ag– cell sizes, respectively, and *T*_*i*,0_ is the initial tumor sizes in *T*_*i*_. We investigated that the total payload differs from that of *β*, followed by a different tumor reduction. Biologically, *T*_1_+*T*_2_ appears to support the idea that, if the Ag+ cells occupy a large proportion, then the TGI effect may be stronger. In addition, the ADC efficacy may depend on the amount of Ag+ cells. Moreover, we may see from *β*=*a*∈(0,1] and *β*=1−*a* that both TGI effects of *T*_1_+*T*_2_ are similar, as shown in Fig. 3c. The total payload *C*_*int,p*_+*C*_*int,n*_ in the tumor cells quickly converges to an equilibrium point. blackThe reason that TGI effects of *T*_1_+*T*_2_ are similar when the ratio of Ag+ cells is *β* and 1−*β* is because the model (2) is the closed system, that is, there is no elimination rate (clearance/volume) of *C*_*ext,p*_. This yields that the total payload *C*_*int,p*_+*C*_*int,n*_ is constant after time elapses (around 1days here) regardless of *β*. Thus, the concentration of *C*_*int,p*_ when an initial size of Ag+ cells is given by *β*·*T*_0_ is approximately similar to *C*_*int,n*_ if the initial Ag+ cell size is (1−*β*)·*T*_0_. We may consider a case *β*→0. Then *C*_*int,p*_→0 and so the tumor cells are only inhibited by bystander killing, but if *β*→1, then *C*_*int,n*_→0 and so the tumor cells are inhibited by direct killing. In addition, if *β*=0, then the concentration of the payload in Ag+ cells should be zero, which cannot define an initial payload *C*_0_. This is the reason to assume *β*>0.

### Bystander-killing effect

Using the developed model, we investigated the influence of *k*, *β*, and the initial payload concentration. The dynamics of the released payload and the tumor growth were observed in separate tumor cells. We next proceeded with capturing the bystander-killing. In addition to the direct killing of Ag+ cells, some of the antibody-drug conjugates (ADCs) also have the capacity to kill adjacent antigen-negative tumor cells, which is called the bystander-killing effect. From the derived payloads *C*_*int,p*_ and *C*_*int,n*_, the bystander-killing effect was explored to capture the additional tumor delay shown in previous known studies [6, 8, 19]. The translated small drug concentration from the Ag+ to Ag– cells may trigger an additional tumor delay. As we discussed earlier, *k* is greater than or equal to 1 because the efflux rate appears to be naturally faster than the influx rate. If the maximum value of *C*_*ext,p*_ is represented as *C*, we then add the first and second equations in (Fig. 1). The rate of the total payload as an inequality is

$$\frac{d\left(C_{int,p}+C_{int,n}\right)}{dt}\leq k_{in}C-k_{out}\left(C_{int,p}+C(int,n)\right).$$

**Fig. 4:**
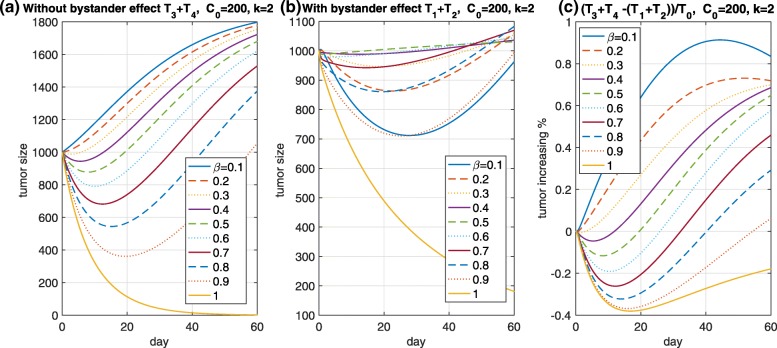
Bystander-killing. *T*_3_ and *T*_4_ represented by the Ag+ and Ag– cells are the tumor sizes without the bystander-killing effect in (**a**). A comparison with/without the bystander-killing effect was investigated in (**b**). The relative change in ratio is presented in (**c**). This indicates that the bystander-killing effect should consider the amount of target-positive cells as well as the payload concentration

This can be calculated and *C*_*int,p*_+*C*_*int,n*_ results in a value of less than or equal to *exp*(−*k*_*out*_*t*)*C*_0_+*C*_0_(1−*exp*(−*k*_*out*_*t*)) because of *k*≥1. Thus, *C*_*int,p*_+*C*_*int,n*_≤*C*_0_, where *C*_0_ is the initial payload concentration in an Ag+ cell. blackThus, it is expected that the TGI effect based on a payload with the bystander-killing effect is weaker than that without it when *β*=1, as shown in Fig. 4a and b (yellow solid curve). Suppose the two cases, “with and without a bystander-killing” whose condition is *k*_*out*_ positive or zero, respectively. Such an ideal case is likely to be reasonable for the following reason: Based on research by Tai et al. [20], monomethyl auristatin E and F (MMAE and MMAF) can be used to determine the bystander-killing of two potent auristatin payloads. Although MMAE and MMAF are structurally similar, MMAF has more hydrophilic molecules and is less membrane permeable than MMAE, which is consistent with its lower membrane permeability. blackAs shown in Fig. 4, if *β*≠1, then bystander-killing is observed. Here, *T*_3_ and *T*_4_ are Ag+ and Ag– cells, respectively, when the bystander-killing effect is off. From Fig. 4a and b, if there is no bystander-killing effect, then the fully Ag+ cells (*β*=1) are the most suppressed, followed by 90% and 80% Ag+ cells in turn. However, if the bystander-killing effect is considered in Fig. 4b, then the dynamics of each tumor in 90% and 10% Ag+ cells among all cells are similar, followed by 80% and 20% Ag+ cells in turn. If the ratio of Ag+ cells to the total cells is higher, then the bystander-killing effect is initially smaller, and an intermediate phase (at approximately zero to 30 days) occurs based on the parameters, i.e., when *β* is smaller, the bystander-killing effect is stronger. This indicates that the efficacy of the ADCs should consider the ratio of Ag+ to Ag– cells. This supports the existence of the bystander-killing effect through the mathematical model. In addition, the extent of the bystander-killing effect is shown in Fig. 4c. As the figure indicates, the ratio of the cells may determine the extent of the bystander-killing effect. Specifically, if *β*=0.7, then the bystander-killing effect will be expected after 30 days under the parameter values and *C*_0_=200. To maximize the tumor treatment when considering the bystander-killing effect, we believe that the amount of intracellular released payload, the ratio of influx rate *k*_*in*_ to efflux rate *k*_*out*_, and *β* should be considered. As a remaining aspect, a direct use of the TGI model appears to be unrealistic, as shown in Fig. 4a and b, because the initial tumor inhibition delayed by the tumor-drug interaction cannot be captured in the initial phase. Thus, a new model should be developed to describe this aspect.

### TGI model used to describe the tumor growth when considering the drug delay

To overcome this problem, we propose an age-structure model [21, 22] based on a non-Markovian stochastic process (see Additional file 1: Appendix). By interacting the payload with the tumor cells, the tumor cells can pass through with abnormal kinetics, which is represented based on the elapsed time. A detailed plan of this is presented as follows. After payload *C* interacts with tumor cell T, T progresses through different stages, which have a time elapse of *a*. Each process has a corresponding age-dependent hazard rate *γ*(*a*), represented by *γ*(*a*)=*ϕ*(*a*)/*Φ*(*a*), where *Φ* is a survival function such that

$$\frac{d\Phi(a)}{da}=-\gamma(a)\Phi(a).$$

The probability density function *ϕ* (pdf) is used to account for the probability that a tumor cell will interact from the eclipse stage into the effect state, which results in a reduction of the tumor cell. To model this stage, assume that *E* and *T* are in *L*^1^, which is a collection of the function *f* such that $\int _{0}^{\infty } f(x)\,dx<\infty $. Applying only the non-Markovian removal process shown in [23], the TGI model is developed as follows.

$$ \frac{dT}{dt}=\int_{0}^{\infty} c\phi(a)E(t-a)F(t-a)T(t-a)\,da-\lambda T. $$

There are many candidates of *ϕ* such as gamma, Weibull, and Mittag-Leffler distributions. The pdf in gamma and Weibull distributions is a combination of the exponential and power function, and the power function and power of the exponential, respectively. It is difficult to find the exact form of the pdf in a Mittag-Leffler distribution, although an age-structure model results in the form of a fractional-order derivative [23]. We first applied a Mittag-Leffler distribution, but the results showed a strong tumor delay for all fractional orders *α*∈(0,1). A Weibull distribution achieved a better result, but the equation is not simple, and the implementation of the simulation took much longer because of the power of the exponential. As a result, we used a gamma distribution for two reasons. It consists of a power function and an exponential, which can describe more phenomena than an exponential alone (Markovian process), and a linear trick may be applied [24, 25], namely, substituting an age-structure model with a system of ODEs. If a probability density function *ϕ* has an Erlang distribution [26], it is considered a special gamma distribution with an integer shape parameter, developed to predict the waiting times in queuing systems [27]. Here,

$$\phi(a)=\frac{1}{\Gamma(n)\theta^{n}}\cdot a^{n-1}e^{-\frac{a}{\theta}},~~n=1,2,3,\cdots,$$

where *θ* is a rate parameter and *n* is a shape parameter. This motivated us to define $F_{\phi }^{n}(t)$ by

$$\int\nolimits_{0}^{\infty} \frac{c}{\Gamma(n)\theta^{n-1}}a^{n-1}e^{-\frac{a}{\theta}}E(t-a)F(t-a)T(t-a)\,da.$$

Note that *θ*^*n*−1^ is used instead of *θ*^*n*^ because of unit consistency. Thus, $F_{\phi }^{n}(t)$ is represented by

$${\begin{aligned} F_{\phi}^{n}(t)=\left\{ \begin{array}{ll} \int_{0}^{t} \frac{c}{\Gamma(n)\theta^{n-1}}a^{n-1}e^{-\frac{a}{\theta}}E(t-a)F(t-a)T(t-a)\,da & \text{for} ~t\geq a\\ \ \ \ \ \ \ \ \ \ \ \ \ \ \ \ 0 & \text{for}~ t< a. \end{array} \right. \end{aligned}} $$

**Fig. 5:**
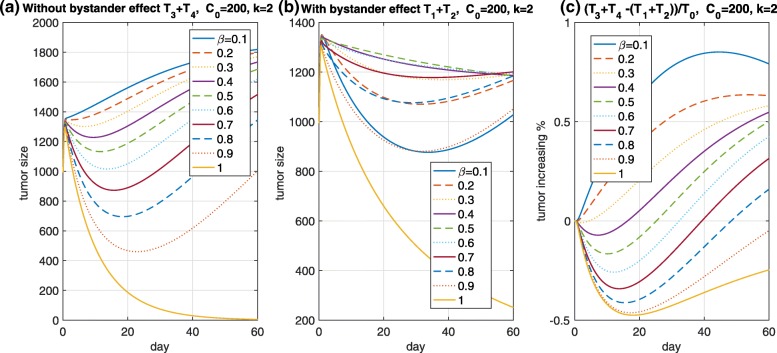
Initial tumor growth. The model based on the age structure shows the tumor inhibition delay for the initial phase at approximately 2 days even though a 200 nM payload is administrated. Here, *T*_1_+*T*_2_ in (**b**) is the total tumor cell size, represented by Ag+ and Ag– cells, and considers the bystander-killing effect from the payload, whereas *T*_3_+*T*_4_ in (**a**) does not consider the bystander-killing effect. The ratio of difference between them to the initial tumor size is shown in (**c**)

By applying a change of variables with *η*=*t*−*a*, and differentiating both sides with respect to *t*, the TGI model is obtained as a system of ODEs. Thus, the TGI model, *T*, is presented as follows.

3 $$ \begin{aligned} \frac{dF_{\phi}^{1}(t)}{dt}=&cE(t)F(t)T(t)-\frac{1}{\theta}F_{\phi}^{1}(t)\\ \frac{dF_{\phi}^{n}(t)}{dt}=&\frac{1}{\theta}\left(F_{\phi}^{n-1}(t)-F_{\phi}^{n}(t)\right)\\ \frac{dT(t)}{dt}=&\frac{1}{\theta}F_{\phi}^{n}(t)-\lambda T(t), \end{aligned}  $$

where $n=2,3,\cdots, F_{\phi }^{1}(0)=cE_{0}T_{0}$, and $F_{\phi }^{n}(0)=0$ for *n*>1. The rate parameter *θ* is chosen as 0.1 days, and the other parameters are the same. An initial tumor growth is observed even if the payload is injected, as shown in Fig. 5. The model is expected to capture a realistic TGI effect. As a result, we propose two new models, one of which is a payload considering two distinct types of cells, and the other is the TGI model, which substitutes the Emax function *E* with the tumor model, based on the age structure.

## Discussion

We explored how an intracellular released payload interacts with the positive and negative target tumor cells. Two tumor cells, Ag+ cells and Ag– cells, were considered. We have investigated that the amount of payload in the Ag+ tumor cells, *k*, and *β* may determine the ADC efficacy. To support this, we proposed that two tumor growth models be applied independently, namely, *T*_1_ and *T*_2_. Otherwise, it is not necessary for TGI models to treat separate tumor cells because the total payload is independent of *β*. Here, *k*=*k*_*out*_/*k*_*in*_ brings about a difference in the payload concentration in Ag+/Ag– cells, as well as their sum. The bystander-killing effect was also examined. Previous studies emphasized that the bystander-killing effect causes an additional tumor reduction during ADC treatment. We observed the difference from the TGI effect through the on/off states of the bystander-killing effect. Finally, a TGI model based on the age-structure using an Erlang distribution enables the tumor inhibition delay to be captured during the initial phase. In addition, the model was transformed into a system of ODEs using a linear trick. Our model suggests a way in which an intracellular-released payload in Ag+ cells can be extracted through lysosomal degradation and mediate the bystander-killing effect, as shown in [6, 28–30]. The models, (2) and (3), are expected to account for the complex ADC dynamics if the mAb-target process is considered. Because mAbs binds reversely to the target antigen in a one-to-one correspondence, a target-mediated drug disposition [31] is likely to be used. In addition, ADC modeling should consider the drug-antibody ratio and the diffusivity from the ADC blood capillary to the tumors, resulting in partial differential equations. This may increase the complexity of the integrated ADC model. As this model does not consider the cleaving extracellularly, the payload concentration is preserved in the tumor cells. Because we did not measure the payload concentration from the ADCs, we may more accurately estimate the bystander-killing if the diffusion rate of the payload-cleaved ADCs and the clearance in the extracellular space are given. To demonstrate the bystander-killing effect in our proposed model, we need to account for the heterogeneous tumor cell environments and explain them independently. Otherwise, capturing may be difficult when the total amounts of the payloads are considered in the tumors. One may consider the case where Ag+ cells are preferentially affected by drug-induced death. This may be realistic in the ADC mechanism. To reflect this in the model, the ADC-target interaction and its internalization process (endocytosis and lysosome) must be considered. If the linker is not rarely broken before ADCs bind to the target and so small amount of the payload diffuses to Ag– cells, then Ag+ cells will be suppressed first. In this case, it is expected that its complex (ADC-target) could be influenced by the target expression level. Therefore, the target concentration may be expressed depending on *β*. In a future study, we plan to develop an integrated model of ADCs that considers the Ag+/Ag– cells and the diffusivity of the payload to demonstrate how the amount of released payload effects the extent of the bystander-killing.

## Conclusions

The bystander-killing effect has been investigated using the released payload in the Ag+ cells. The proportion of *β* determines amount of Ag+/Ag– cells and the change of the tumor sizes by the proportion of *β* is applied in the TGI model independently for capturing bystander killing. Also, we found that the TGI model based on the age-structure may describe the initial delay through the drug-tumor interaction.

## Additional file

Additional file 1 contains Appendix for a survival function and development of an age-structure model related to the TGI model in the main body of the paper. (PDF 130 kb)

## Abbreviations

ADCs: Antibody-drug conjugates
Ag-: Antigen negative
Ag+: Antigen positive
DM1: Emtansine
Eq.: Equation
Fig.: Figure
MMAE: Monomethyl auristatin E
TGI: Tumor growth inhibition
