# Supplementary material for: Modeling to capture bystander-killing effect by released payload in target positive tumor cells
Source: BMC Cancer. 2019 Mar 4;19:194. doi: 10.1186/s12885-019-5336-7 (PMC6399851; doi:10.1186/s12885-019-5336-7)
Supplement: Supplementary file 1 — contains Appendix for a survival function and development of an age-structure model related to the TGI model in the main body of the paper. (PDF 130 kb) [file 12885_2019_5336_MOESM1_ESM.pdf]

Appendix

Survival function

A stochastic model may be defined based on its retention-time distributions. It relies on a continuous-time probability distribution rather than on a conditional transfer probability in discretized units of size Δt. Let A be Ag- cells of a particle in a compartment. Define a cumulative distribution function $F(a)$, i.e., $F(a) = Pr(A < a)$. Then, create a density function ϕ defined by $\phi(a) = dF(a)/da$. Note that $F(a) = Pr(A < a)$ is actually the distribution function of A, which indicates the probability that a molecule will leave the compartment prior to attaining Ag- cells a. We can denote the survival function Φ as $\Phi(a) = P(A \geq a) = 1 - F(a)$ to represent the probability that the molecule survives in the compartment to reach the Ag- cells by a. Define a hazard function $h(a)$ as

$$h(a) = \frac{\phi(a)}{\Phi(a)}.$$

This yields a simple relationship

$$\frac{dlog\Phi(a)}{da} = -h(a),$$

which explains the relation between the hazard rate and the survival function.

Development of an age-structure model

Let $\rho(t, a)$ be a flux density of an ensemble of particles that have reached age a in the compartment at time t. It is assumed that the density of the particles created in the compartment by the creation process between t and $t + \Delta t$ is $\beta(t)\Delta t + o(\Delta t)$. Note that based on continuous time random walk, the creation process is a removal process from a previous compartment. We consider that the probability of a particle being removed from the ensemble at time t only depends on the state of the system at time t. A particle will be removed through the Markovian removal process from t to $t + \Delta t$, which is $\lambda(t)\Delta t + o(\Delta t)$. Then, a survival function is as follows:

$$\Theta(t, t_0) = e^{-\int_{t_0}^{t} \lambda(s)\, ds}. \tag{1}$$

We also consider a non-Markovian removal process, in which the probability that a particle is removed from the ensemble depends on the length of time after the particle has entered the compartment. If the particles enter the compartment at t_0 and survive until age $a = t - t_0$, then the survival function for a non-Markovian removal process is given by

$$\Phi(a) = 1 - \int_{t_0}^{t} \phi(u)\, du, \tag{2}$$

where ϕ is the probability density function such that $\int_{t_0}^{t} \phi(u)\, du$ is the probability that the particle leaves the compartment before time t. If we consider

$q(t)$ as the flux into the compartment at t-based on CTRW, then $q(t) = q_0\delta(t-0+) + q^+(t)$, where δ is derac-delta, q_0 is a constant, and q^+ is right continuous at $t = 0$, and continuous at $t > 0$. If the Markovian removal process and non-Markovian removal process are independent, we may define $\rho(t,a)$ as

$$\rho(t,a) = \Phi(a)\Theta(t,t-a)q(t-a),$$

with $\rho(t,0) = q(t)$. Then, the age-structure equation can now be found by differentiating with respect to time. This can be expressed as

$$\begin{aligned}\frac{\partial \rho}{\partial t} + \frac{\partial \rho}{\partial a} = &\frac{d\Phi(a)}{da}\Theta(t,t-a)q(t-a)\\ &+ \Phi(a)q(t-a)\Big(\frac{\partial \Theta(t,t-a)}{\partial t} + \frac{\partial \Theta(t,t-a)}{\partial a}\Big),\end{aligned} \tag{3}$$

where we use the relations in which $\frac{dt}{dt} = \frac{da}{dt} = 1$ and $\frac{\partial q(t-a)}{\partial t} = -\frac{\partial q(t-a)}{\partial a}$. If we define a hazard rate γ dependent only on age a by $\gamma(a) = \frac{\phi(t)}{\Phi(a)}$ as [**?**], then the rate of change of Φ is as follows:

$$\frac{d\Phi(a)}{da} = -\gamma(a)\Phi(a), \tag{4}$$

by $\frac{d\Phi(a)}{da} = -\phi(a)$. The elementary equation (4) is solved as

$$\Phi(a) = e^{-\int_0^a \gamma(s)\,ds},$$

which equals (2). Note that the Markovian survival function is given by (1)

$$\Theta(t,t-a) = e^{-\int_{t-a}^t \lambda(s)\,ds}. \tag{5}$$

With all things considered above, (5) is

$$\begin{aligned}\frac{\partial \Theta(t,t-a)}{\partial t} + \frac{\partial \Theta(t,t-a)}{\partial a} &= -(\lambda(t) - \lambda(t-a))\Theta(t,t-a) - \lambda(t-a)\Theta(t,t-a)\\ &= -\lambda(t)\Theta(t,t-a)\end{aligned} \tag{6}$$

and substituting (4) and (6) into (3), we have

$$\frac{\partial \rho(t,a)}{\partial t} + \frac{\partial \rho(t,a)}{\partial a} = -\gamma(a)\rho(t,a) - \lambda(t)\rho(t,a), \tag{7}$$

where $\rho(t,0) = q(t)$. Summing all ages, $\rho(t) = \int_0^t \rho(t,a)\,da$. Then,

$$\frac{d\rho}{dt} = \rho(t,t) + \int_0^t \frac{\partial \rho(t,a)}{\partial t}\,da. \tag{8}$$

Integrating (7) with respect to a,

$$\int_0^t \frac{\partial \rho(t,a)}{\partial t}\,da + \rho(t,t) - \rho(t,0) = -\lambda(t)\rho(t) - \int_0^t \gamma(a)\rho(t,a)\,da. \tag{9}$$

Substituting (9) into (8),

$$\begin{aligned}\frac{d\rho(t)}{dt} &= \rho(t,t) - \rho(t,t) + \rho(t,0) - \lambda(t)\rho(t) - \int_0^t \gamma(a)\rho(t,a)\,da \\ &= q^+(t) - \lambda(t)\rho(t) - \int_0^t \gamma(a)\Phi(a)\Theta(t,t-a)q(t-a)\,da.\end{aligned} \tag{10}$$

Because $\gamma(a) = \frac{\phi(a)}{\Phi(a)}$, (10) becomes

$$\frac{d\rho(t)}{dt} = q^+(t) - \lambda(t)\rho(t) - \int_0^t \phi(a)\Theta(t,t-a)q(t-a)\,da.$$

This is an evolution equation of the compartment when considering both Markovian and non-Markovian processes. For a multi-compartment case, we may define the equation as follows.

$$\begin{aligned}\frac{d\rho_k(t)}{dt} &= q_k^+(t) - \lambda_k(t)\rho_k(t) \\ &\quad - \int_0^t \phi_k(a)\Theta_k(t,t-a)q_k(t-a)\,da, \quad k = 1, 2, \cdots, m \in N.\end{aligned}$$
